# Annotations

**Published:** 1888-12-08

**Authors:** 


### ANNOTATIONS.

**The Slaughter of Shop-Assistants.**

It cannot be denied that Britons have a great capacity for slaughter. Whether it be savages or small birds, pheasants, foxes, or shop-assistants it makes little difference—slaughter we do and slaughter we apparently intend to do. How many years has the association of which Sir John Lubbock is the patron and guide been in existence and in active operation? And yet what results of a practical character has it accomplished? It cannot be pretended that employers of shop-assistants have not the brains to understand that too many hours in a close atmosphere every day means disease and premature death; nor can it for a moment be believed that they have not sense enough to know that the public have certain wants, and that they will supply those wants just as fully if they are compelled to do it in eight hours as they will if they are permitted to spend eighteen in the process. The master shopkeepers, we say, have brains enough to understand these simple problems. If they have not, can it be affirmed with any truth that they have any brains at all? But what do we find? This: That in many parts of the United Kingdom the working hours of shop-assistants still number as many as 84 or 86 per week—fourteen hours of work every day for six days a week. When the fact was announced at Willis's Rooms last week somebody cried out "Shame!" He should have cried out "Pillory!" "Shame" is out of date; nobody understands or feels it nowadays. Our forefathers had ruder, but more effectual methods of dealing with creatures whose kinship with the lower animals was so near that they had not developed either conscience or reason. They put them into the pillory and treated them to a liberal regimen of eggs, not fresh, applied externally. We do not advise that as a first step towards the creation of a more stimulating environment. An experimental effort might be made by publishing the names of those stupid and selfish shopkeepers who are so ready to sacrifice all whom they may employ at the shrine of their own prosperity. If this mild form of "pillory" should prove insufficient, there seems to be no good reason why we should not revert to some of the methods of earlier times. Advanced civilisation may be a very good thing, but it is only to be appreciated by advanced intelligence and an educated conscience. Persons of character may be admonished by reproof; murderers have to be admonished by the hangman. Shopkeepers who keep open too long are not murderers, inasmuch as they have no intention of murder in their hearts. But they have the fixed intention of making money, even at the expense of incalculable injury to others. That intention should be recognised, and if public opinion is not sufficient to prevent it issuing in injury to others, undoutedly the State should interfere and protect its citizens by adequate measures of prohibition and punishment.

**The State and the Shop.**

It would take reams of paper and many lifetimes to set forth in detail what the State is. What it ought to be may be condensed into a single sentence: The State should be the quintessence of the best judgment of the time in unrestricted action. Has this rare essence ever been turned upon the woes of shop-assistants? We say "woes," for the word is no whit too strong for the case. Every medical man of any experience in middle-class practice must know the condition of these people, especially of the young women. How many of them are so bloodless that they can neither eat nor digest? As a result of their bloodlessness they are not fit either to stand behind the counter or to lift weights from the shelves. At least a third, perhaps half, of the shopgirls in large towns are in such a wretched state of health that the only proper thing to be done with them would be to send them straight off to the seaside for six months; to ply them with steel during the greater part of that time, and to feed them on the very best of convalescent diet. In six months they would begin to look healthy. Now the Englishwoman is naturally a very fine, handsome, and spirited specimen of her sex. The class from which shop-women are drawn – farmers and tradesmen of the middle rank —is one of the healthiest and most vigorous of our population. The girls, before their entrance upon shop life, are usually selected with a good deal of care, because they must have a certain superiority of physique and personal appearance in order to be suitable for the business at all. Here, then, we take the pick and flower of our lower middle classes, and utterly destroy them in health and physique. But most of these girls ultimately become wives and mothers. What kind of mothers can they be? Bloodless, strengthless, with both brain and body reduced to the lowest level compatible with a feeble existence, they are fitter to be museum specimens of nineteenth century stupidity than to be wives and mothers. Sir John Lubbock said at his meeting that they were told "these evils ought to be cured by voluntary action. They had been working on voluntary lines for years, and how much longer were they to do so? How many more generations of shop assistants were to be sacrificed before they took a bold stand?" Undoubtedly Sir John Lubbock was right. No result at all commensurate with the magnitude of the evil will ever come from voluntary action. The only voluntary action possible to a wolf who happens to meet a nice fat lamb in a quiet place, is that of eating him up. According to the gospel of political economy, which in this particular is amply borne out by ages of experience, the only voluntary action possible to the shopkeeper is that of doing his utmost, by all possible means, to secure his normal profit of five, fifteen, or twenty-five per cent. The whole State is the natural protector of each of its members. If it be not, what is the use of any State life at all? If political economy insists that everything must be left to the play of the natural forces, then political economy reduces us to the level of a pack of wolves or any other gregarious animals. The State must find out ways and means to protect the weak against the strong; otherwise all money spent by the State is pure waste; and the State itself is a worthless sham.

**Washing Bills: A Suggestion.**

A correspondent suggests that the laundry work of the general hospitals might very well be performed by prisoners or paupers. She has given some years to the consideration of the question, and has not been able to discover any fatal or even serious objection. The scheme would be advantageous to hospitals in the very considerable saving of money which would result. It is not, however, quite so simple as it may at first sight appear. Hospitals are, of course, not State institutions, but voluntary charities. There is, therefore, no more reason why prisons or poorhouses should do their washing than why they should wash for blind asylums, orphanages, or other charitable organisations. In the case of prisoners, the work could only be imposed as a punishment like other hard labour. Prisoners have not hitherto, at least to any large extent, been employed in productive labour in the open market. At the Wakefield prison mat-making has been long a prosperous industry; but many people object to the payment of prison rates which help prison officials to undersell them in their own markets. Prisoners should, no doubt, be employed, and always, when possible, in productive labour, But it seems best on the whole that production should be limited to articles of all kinds which are needed in prisons themselves. Prison labour should not compete with open market labour. As far as paupers are concerned, they may very properly object to being classed with prisoners. The same objections, too, which apply to the productions of prison labour being sold in the open market apply here. Paupers may, and ought to produce all they can for themselves, and thus save the ratepayers pocket. But when it comes to entering into business competition with ratepayers, the question assumes a totally different complexion. By all means let prisons and poorhouses be made as nearly self-supporting as possible; but prison labour and pauper labour must not be allowed to make the poor man's earnings and the employers meagre profits still smaller than they are already. Hospitals, it is to be feared, must continue to bear the very serious burdens of theirown washing bills.

